# A Curious Story in Relation to Hereditary Suicide

**Published:** 1898-02

**Authors:** 


A CURIOUS story in relation to hereditary suicide comes to us from Paris, which we give on the authority of Professor Brouardel. A farmer killed himself by hanging, leaving seven sons and four daughters. Ten of these eleven children, after they had each brought up families, took their own lives, and their several offsprings also committed suicide by various means and at various ages. The remaining survivor of the original eleven children is now a man sixty-eight years, and is supposed to have passed the probable age of his family's suicidal tendencies—at any rate, it is only charitable to assume so.—*Health.*

